# Optimization of the Medium for the Production of Extracellular Amylase by the* Pseudomonas stutzeri* ISL B5 Isolated from Municipal Solid Waste

**DOI:** 10.1155/2016/4950743

**Published:** 2016-12-20

**Authors:** Prajesh Dutta, Akash Deb, Sukanta Majumdar

**Affiliations:** Microbiology and Microbial Biotechnology Laboratory, Department of Botany, University of Gour Banga, Malda, West Bengal 732103, India

## Abstract

The management of municipal solid waste is one of the major problems of the present world. The use of microbial enzymes for sustainable management of the solid waste is the need of the time. In the present study, we have isolated a potent amylase producing strain (ISL B5) from municipal solid waste. The strain was identified as* Pseudomonas stutzeri (P. stutzeri)* both biochemically and by 16S rDNA sequencing. The optimization studies revealed that the strain ISL B5 exhibited maximum activity in the liquid media containing 2% starch (2.77 U/ml), 0.8% peptone (2.77 U/ml), and 0.001% Ca^2+^ ion (2.49 U/ml) under the pH 7.5 (2.59 U/ml), temperature 40°C (2.63 U/ml), and 25 h of incubation period (2.49 U/ml). The highest activity of crude enzyme has also been optimized at the pH 8 (2.49 U/ml).

## 1. Introduction

Microorganisms are most important sources of enzyme production which can be used for various purposes of humans. Microbial enzymes have several advantages, which comprise lower production costs, possibility of large-scale production in industrial fermenters, wide range of physical and chemical characteristics, scope of genetic manipulation, and rapid culture development [1]. The enzymes produced by microorganisms are also more active and stable than plant and animal counterparts [2]. The above characteristics make microbial enzymes suitable for various industrial applications [3]. In addition, as the microorganisms can be cultured in large quantities in a short time by fermentation, they represent an alternative source of enzymes. Presently, microbial enzymes are considered to be increasingly important for sustainable technology and green chemistry [2, 4].

Amylase is one of the important enzymes, used in the field of biotechnology. It performs the hydrolysis of starch to yield glucose [5]. In recent years, the microbial production has made its superiority due to its wide spread use in food, baking, and detergent and textile industries [6]. There are so many advantages of using microorganisms for their ability in mass production of amylase and also for their very easy manipulation for desired products [7]. *α* -Amylase has been derived from many fungi, yeasts, bacteria, and actinomycetes; however, enzymes from fungal and bacterial sources have been considered most suitable for applications in industrial sectors [8]. Several microorganisms are able to make amylases including* Bacillus* spp.,* Lactobacillus*,* Pseudomonas* sp.,* Proteus*,* Escherichia*, and* Streptomyces* sp. [9].

Municipal solid waste management is tremendous problem in front of current world. One of the sustainable management of solid wastes is to digest it to produce an end product which can be used as a resource. Several products, including biofertilizers, have been reported to be produced from municipal solid waste. Several reports suggested that municipal solid waste can be transformed into biofertilizer with multifunctional efficiency. Biofertilizer generated from municipal solid waste are rich in microorganisms with various capabilities as these products are generated from microbial action where microbes use various substrates like starch, cellulose, and proteins. Composting is one of the methods of converting organic wastes into biofertilizers, reducing the inorganic compound usage that may lead to the environmental contamination. This conversion is the consequence of the action of microorganism, transforming complex carbon sources into resultant energy. Production of enzymes by microbes to its environment leads this process [10].

The present study focuses on the search of the starchy material transforming capacity of amylolytic bacteria present in the municipal waste. For the fulfilment of this objective isolation, characterization and identification of amylolytic bacteria and partial characterization of amylase enzyme with regard to the effect of substrate, temperature, and pH were done.

## 2. Materials and Methods

### 2.1. Sample Collection

In this study for the purpose of isolation of amylase producers, soil samples were collected from the municipal solid waste deposition area of Malda town, West Bengal, India. The soil samples were collected in sterilized polyethylene bags and brought in ice pack to the laboratory.

### 2.2. Isolation of Amylase Producing Bacteria

One gram (1 g) of the soil sample was weighed and added to 9 ml of sterile distilled water. Serial dilutions were prepared up to the 10^−4^ dilution and then 0.1 ml of each dilution was added, using the spread plate method, to nutrient agar that had been fortified with 1% starch. The agar plates were incubated at 37°C for 24–48 h and then flooded with Lugol's iodine. The colonies produced halo zones were designated as amylase producers, picked, and maintained in NA slants supplemented with 1% starch.

### 2.3. Characterization and Identification

The bacterial isolates were characterized based on the following morphological and biochemical tests such as Gram staining, scanning electron microscopy (SEM) using Hitachi Scanning Electron Microscopes (model S-530), catalase test, production of acid and gas from carbohydrate, nitrate reduction, protein hydrolysis, gelatin liquefaction, and Voges-Proskauer (VP) test [11, 12].

The strain was identified by both biochemical and molecular approaches. Biochemically, the stain was identified by using the BiomerieusVitek 2 system.

For molecular identification, genomic DNA was extracted from 24-hour-old culture following the method of Stafford et al. [13]. DNA was precipitated from the aqueous phase with chilled ethanol (100%) and pelleted by centrifuging at 12000 rpm for 15 min, followed by washing in 70% ethanol and centrifugation. The pellets were air-dried and suspended in TE buffer pH 8.

For PCR amplification, DNA was amplified by mixing the template DNA (50 ng) with the polymerase reaction buffer, dNTP mix, primers, and Taq polymerase. Polymerase chain reaction was performed in a total volume of 100 *μ*l, containing 78 *μ*l deionized water, 10 *μ*l 10x Taq polymerase buffer, 1 *μ*l of 1U Taq polymerase, 6 *μ*l 2 mM dNTPs, 1.5 *μ*l of 100 mM reverse and forward primers, and 3.5 *μ*l of 50 ng template DNA. The amplification of 16S rRNA gene was carried out by PCR using the forward (704F 5′GTAGCGGTGAAATGCGTAGA 3′) and reverse** (**907R 5′CCGTCAATTCMTTTGAGTTTAG 3′) primer. The PCR was programmed with an initial denaturing at 94°C for 5 min, followed by 30 cycles of denaturation at 94°C for 30 sec, annealing at 61°C for 30 sec, and extension at 70°C for 2 min and with a final extension at 72°C for 7 min in a thermocycler (Applied Biosystems, 2720). Amplified products were resolved by electrophoresis in 0.8% agarose gel and PCR amplicons were purified. The purified DNA was sequenced from Xcelris laboratories, Ahmadabad, India, and the 16S rDNA sequence obtained from PCR products was subjected to BLAST analyses. The DNA sequences were deposited to NCBI GenBank through BankIt procedure and approved as the sequence after complete annotation and given accession numbers. Evolutionary history was inferred by neighbor-joining method [14]. Phylogenetic analyses were conducted in MEGA 4.0 software [15].

### 2.4. Assay of Amylase

For assay, previously inoculated nutrient starch broth was centrifuged at 8000*g*, for 12 minutes, and the supernatant was used as crude enzyme source. The assay of amylase was conducted following the method of Jamieson et al. [16]. In brief, one ml of diluted enzyme solution was added to 1 ml of substrate and then incubated for three minutes at 37°C; two ml of color reagent was added to stop the enzyme reaction; tubes were heated in a boiling water bath for five minutes to effect the color change and then cooled with running tap water and absorbance was read in a spectrophotometer at 470 nm of spectrophotometer [17]. Units of amylase activity were expressed as micromoles of maltose liberated per minute.

### 2.5. Optimization of Amylase Production

The effect of different parameters on the amylase production by the isolate was standardized in respect of incubation time, temperature, pH, carbon source, nitrogen source, and metal ions.

### 2.6. Effect of Incubation Period

After inoculation, the flasks were incubated at 35°C for different time periods ranging from 5 hours to 30 hours.

### 2.7. Effect of Temperature

Effect of temperature on amylase production was studied in the nutrient starch broth at different temperature (28°C to 48°C).

### 2.8. Effect of pH

The amylase production in relation to initial medium pH was studied by inoculating the bacteria in nutrient starch broth, by adjusting the pH ranging from 5.0 to 8.0.

Effect of pH of reaction mixture on amylase production was tested by using buffer (0.1 M) of different pH. By using sodium phosphate (pH 6), potassium phosphate (pH 7), tris-HCl (pH 8), and glycine–NaOH (pH 10) buffers, different pH of the reaction mixture was maintained during the enzyme assay [18, 19].

### 2.9. Effect of Carbon Source

The effect of various carbon sources such as starch, sucrose, glucose, and mannitol at the concentration of 2% was examined for amylase production. The effect of starch concentration on amylase production was determined by supplementing the nutrient broth with different concentration of starch ranging from 0.2% to 4%.

### 2.10. Effect of Nitrogen Source

The effects of nitrogen sources on amylase production were determined by using different organic and inorganic nitrogen sources (0.6%) such as beef extract, ammonium chloride, peptone, and tryptone. The effect of peptone at varied concentration on amylase production was checked by supplementing the nutrient starch broth with different concentration of peptone, ranging from 0.3 to 2.0%.

### 2.11. Effect of Metal Ions

Effects of metal ions on amylase production were checked by substituting different metal ions, ferrous ions, zinc, manganese, and calcium, at 0.001% concentration with the nutrient starch broth [20].

### 2.12. Statistical Analysis

All the optimization studies were conducted in triplicate and the data were analyzed using one-way analysis of variance (ANOVA). All the data are graphically presented as mean ± SD of triplicates (*n* = 3). ANOVA was performed using SPSS software.* P* values < 0.05 were considered significant with a confidence limit of 95%.

## 3. Results

A total of twenty-five strains were isolated with amylase activity. Among them, the isolate ISL B5 showed the highest zone of clearance around the colony when flooded with Lugol's iodine solution. The isolate ISL B5 was characterized both morphologically and biochemically. Light microscopic observation revealed that the isolate was a rod shaped Gram negative bacteria. The morphology of the isolate was also confirmed by scanning electron microscopy.

**Figure 1:**
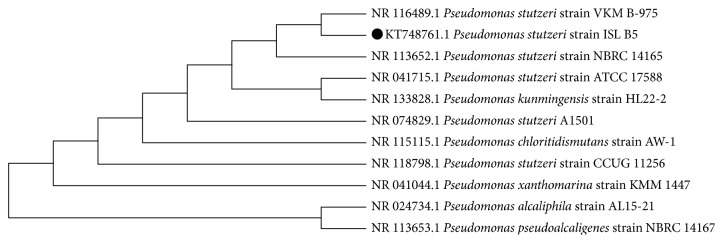
Phylogenetic analysis of 16S rDNA sequences of* P. Stutzeri* ISL B5 (KT748761) with other ex-type strains by neighbor-joining method.

The identity of the isolate was confirmed by both biochemical and molecular techniques. Biochemically, the strain was identified by using the BiomerieusVitek 2 system as* Pseudomonas stutzeri* with 99% probability. Molecular analysis based on 16S rDNA gene homology identified the ISL B5 as* Pseudomonas stutzeri* with 99% similarity with the respective strains in NCBI GenBank database with query coverage of 95%. The obtained sequence was aligned with ex-type isolate sequences from NCBI GenBank for identification as well as studying phylogenetic relationship with other ex-type sequences (Figure 1). The evolutionary distances were computed using the Maximum Composite Likelihood method [21, 22]. The nucleotide sequences were deposited in NCBI GenBank database under accession number KT748761.

**Figure 2:**
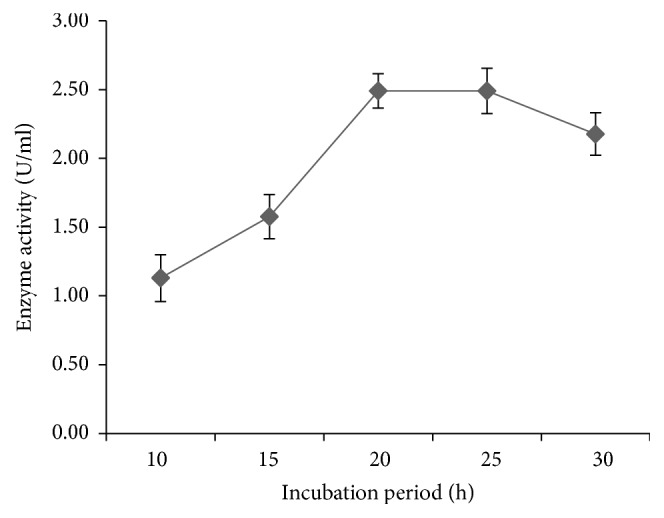
Effect of incubation period on amylase production by* P. stutzeri* ISL B5. Data represent mean ± SD (*n* = 3).

The amylase production by* P. stutzeri* ISL B5 was optimized in terms of incubation period, temperature, pH, carbon and nitrogen source, and metal irons. After inoculation, the flasks were incubated at 30°C and enzyme activity was measured at different time intervals (Figure 2). The isolate showed highest production of amylase after 25 hours of incubation (2.49 U/ml). The yield of enzyme decreased after 25 hours maybe due to the decrease in growth of the isolate.

**Figure 3:**
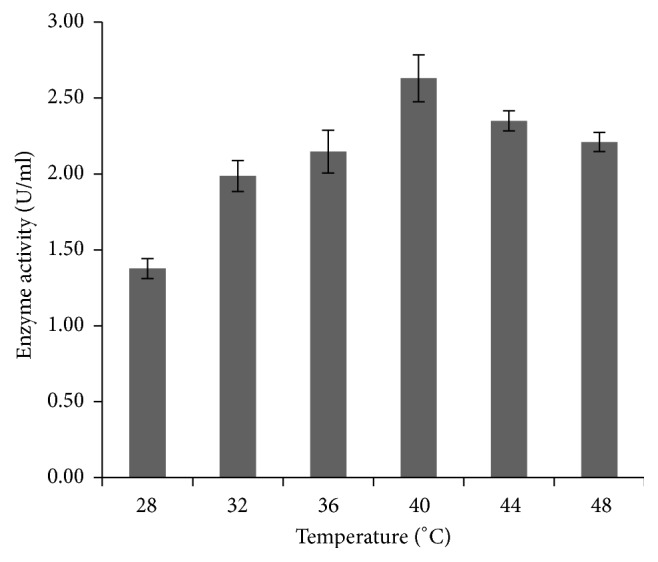
Effect of temperature on amylase production by* P. stutzeri* ISL B5. Data represent mean ± SD (*n* = 3); *P* < 0.05.

The effect of temperature on enzyme production was assessed by maintaining the flasks at different temperature ranging from 28°C to 48°C for 25 h (Figure 3). The maximum enzyme production was detected at 40°C (2.63 U/ml). The enzyme production was declined below and above 40°C temperature.

**Figure 4:**
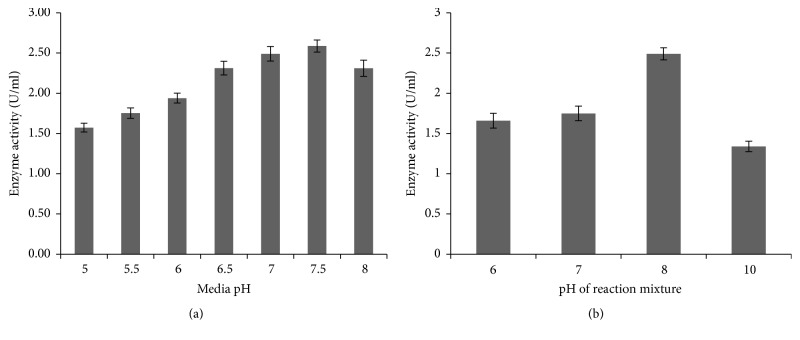
Effect of pH on amylase production by* P. stutzeri* ISL B5 and on enzyme activity in reaction mixture. (a) Amylase production under different media pH. (b) Enzyme activity under different pH of reaction mixture. Data represent mean ± SD (*n* = 3); *P* < 0.05.

The media pH was adjusted from 5.0 to 8.0 for the assessment of amylase production (Figure 4(a)). After 25 h of incubation, it was observed that, in pH 7.5, enzyme was produced maximally (2.59 U/ml) by ISL B5 strain. Effect of pH of reaction mixture on amylase production was also tested by using sodium phosphate (pH 6), potassium phosphate (pH 7), tris-HCl (pH 8), and glycine–NaOH (pH 10) buffers during the enzyme assay (Figure 4(b)) [18, 19]. It was observed that, after 25 hours of incubation, the reaction mixture containing tris-HCl buffer (pH 8) showed maximum amylase production (2.49 U/ml).

**Figure 5:**
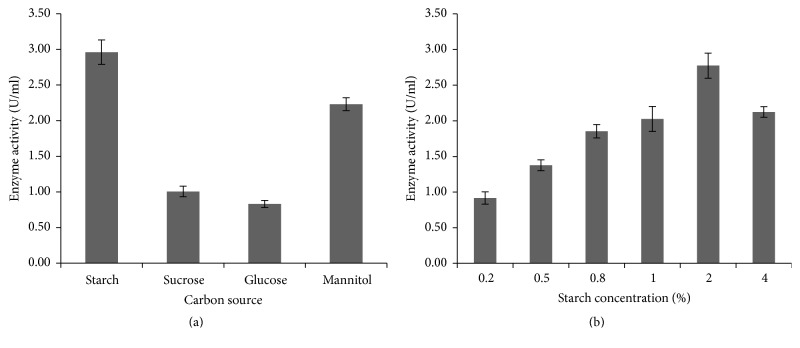
Effect different carbon sources and different starch concentrations on amylase production by* P. stutzeri* ISL B5. (a) Amylase production under the influence of different carbon sources. (b) Amylase production under the influence of different starch concentrations in percentage. Data represent mean ± SD (*n* = 3); *P* < 0.05.

The bacterial isolate ISL B5 was inoculated in nutrient broth, containing starch, sucrose, glucose, and mannitol, to show the effect of carbon sources in amylase production (Figure 5(a)). The starch showed the highest enzyme production at 2% concentration (2.77 U/ml) (Figure 5(b)).

**Figure 6:**
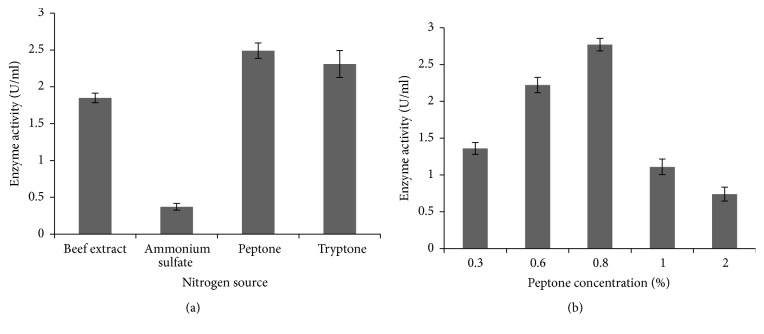
Effect different nitrogen sources and different peptone concentrations on amylase production by* P. stutzeri* ISL B5. (a) Amylase production under the influence of different nitrogen sources. (b) Amylase production under the influence of different peptone concentrations in percentage. Data represent mean ± SD (*n* = 3); *P* < 0.05.

The effect of different nitrogen sources on amylase production was assessed by using beef extract, ammonium chloride, peptone, and tryptone. The maximum enzyme production was exhibited in 0.8% peptone concentration (2.77 U/ml), whereas ammonium chloride had the lowest enzyme production ability (Figures 6(a) and 6(b)).

**Figure 7:**
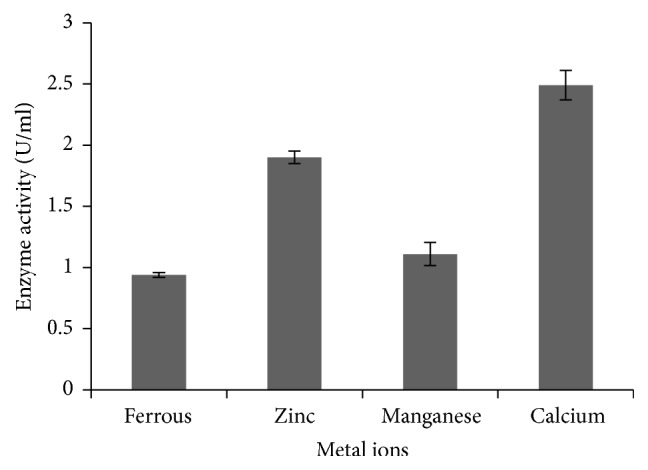
Effect different metal ions on amylase production by* P. stutzeri* ISL B5. Data represent mean ± SD (*n* = 3); *P* < 0.05.

Ferrous, zinc, manganese, and calcium ions in very low concentration were used to determine the effect of metal ions on amylase production (Figure 7). After 25 hours, broth containing calcium ion showed the highest ability of enzyme production (2.49 U/ml), whereas ferrous ion had the lowest ability of enzyme production.

## 4. Discussion

Twenty-five bacterial strains were isolated from municipal dumping waste; from these, the isolate ISL B5, which was identified as* Pseudomonas stutzeri,* showed the highest amylase activity. Several reports have suggested that many bacteria isolated from solid waste show amylase activity with significant efficiency [23]. Among bacterial isolates,* Bacillus* sp. [24] and* Pseudomonas* sp. [25] are frequent amylase producers.

*P. stutzeri* ISL B5 showed the highest amylase production at 25 hours of incubation. Above this incubation period, the amylase enzyme activity started to decrease. This may be due to the decrease in growth of the isolate. Most of the studies reported the highest enzyme production between 35 hours and 48 hours [26, 27]; on the contrary, ISL B5 showed optimum production after 25 hours, thus proving early harvesting time for industrial use.

The strain of* P. stutzeri* has low starch degrading activity below and above 40°C. This may be due to decreased growth rate and inactivation of genes, which are responsible for the starch degrading enzyme [28]. Most of the amylase producing bacterial strain exhibited a pH range between 6.0 and 7.5 for normal growth and enzyme production [29]. The present bacterial strain revealed maximal enzyme production at pH 7.5. The highest enzyme activity in reaction mixture has been achieved at pH 8. Samanta et al. [30] also reported the highest amylase activity of* Cronobacter sakazakii Jor*52 at pH 8.

The supplement of carbon sources in either monosaccharide or polysaccharide form may induce the amylase production. In our present study, the influence of starch was more than the other carbon sources tested. Mannitol was the second best supplementary carbon source. Glucose has the lowest amylase activity. It is reported that the different carbon sources variedly influence the amylase production [31]. Similar findings suggested that glucose represses the amylase production in the case of hyperthermophilic archaea* Sulfolobus solfataricus* [32]. They also reported that glucose inhibits the expression of amylase gene.

The nitrogen sources are secondary energy sources for the organisms, and those play an important role in the growth and in the production of valuable enzymes of organisms. The nature of the compound and the concentration that we used might influence or downregulate enzyme production [27]. In this experiment, the effect of nitrogen sources on amylase production showed that peptone was found to be a better nitrogen source for* P. stutzeri* ISL B5.

The effects of metal ions have been well studied on several amylases from bacteria and fungi. It has been known that most of amylases are metal ion-dependent enzymes and these ions are divalent cations such as Mn^2+^, Zn^2+^, Mg^2+^, Ca^2+^, and Fe^2+^ [17]. Enhancement of amylase activity in the presence of ions could be based on its ability to interact with negatively charged amino acid residues such as aspartic and glutamic acid [33]. The study showed the highest enzyme activity in the presence of calcium ion. According to Burhan et al. [34], in case of* Bacillus *sp., calcium ion increased the production of amylase. Ramesh and Lonsane [35] also reported that different concentrations of calcium affect the amylase production and its activity. The crystal structure of amylase showed that calcium ions play a detrimental role in ionic interaction with Asn 100 and His 201 residues of domain A and also with Asp 159 and Asp 167 residues of domain B in amylase enzyme. It has also been reported that the active site of amylase is located between domain A and domain B and calcium promotes stability and catalytic activity of the enzyme by interconnecting these two domains [36, 37].

In the present study, we have isolated and identified amylase producing bacteria from the municipal solid waste. Our study showed that the municipal solid wastes can be used as productive sources of beneficial microbes; those can be successfully used in large scale for management of municipal starchy waste materials present in municipal waste. The isolate* Pseudomonas stutzeri* ISL B5 showed the ability to tolerate adverse conditions like wide range of pH and temperature and has significant toxic metal ion tolerance, which makes it a promising inoculant for enzyme industry as well as in solid waste management.

## References

[1] Duza B., Mastan S. A. (2013). Microbial enzymes and their applications-a review. *Indo American Journal of Pharmaceutical Research*.

[2] Anbu P., Gopinath S. C. B., Cihan A. C., Chaulagain B. P. (2013). Microbial enzymes and their applications in industries and medicine. *BioMed Research International*.

[3] Hasan F., Shah A. A., Hameed A. (2006). Industrial applications of microbial lipases. *Enzyme and Microbial Technology*.

[4] Kiro M. (2012). Microbial *α*-amylases and their industrial applications: a review. *International Journals of Management, IT and Engineering*.

[5] Omemu A. M., Akpan I., Bankole M. O., Teniola O. D. (2005). Hydrolysis of raw tuber starches by amylase of Aspergillus niger AM07 isolated from the soil. *African Journal of Biotechnology*.

[6] Verma V., Avasthi M. S., Gupta A. R., Singh M., Kushwaha A. (2012). Amylase production and purification from bacteria isolated from a waste potato dumpsite in district Farrukhabad U.P state India. *European Journal of Experimental Biology*.

[7] Lonsane B. K., Ramesh M. V. (1990). Production of bacterial thermostable *α*-amylase by solid-state fermentation: a potential tool for achieving economy in enzyme production and starch hydrolysis. *Advances in Applied Microbiology*.

[8] Manonmani H. K., Kunhi A. A. M. (1999). Interference of thiol-compounds with dextrinizing activity assay of *α*-amylase by starch-iodine colour reaction: modification of the method to eliminate this interference. *World Journal of Microbiology and Biotechnology*.

[9] Parmar D., Pandya A. (2012). Characterization of amylase producing bacterial isolates. *Bulletin of Environment, Pharmacology and Life Sciences*.

[10] Pascon R. C., Bergamo R. F., Spinelli R. X. (2011). Amylolytic microorganism from São Paulo zoo composting: isolation, identification, and amylase production. *Enzyme Research*.

[11] Rohban R., Amoozegar M. A., Ventosa A. (2009). Screening and isolation of halophilic bacteria producing extracellular hydrolyses from Howz Soltan Lake, Iran. *Journal of Industrial Microbiology and Biotechnology*.

[12] Gangadharan D., Madhavan Nampoothiri K., Sivaramakrishnan S., Pandey A. (2009). Immobilized bacterial *α*-amylase for effective hydrolysis of raw and soluble starch. *Food Research International*.

[13] Stafford W. H. L., Baker G. C., Brown S. A., Burton S. G., Cowan D. A. (2005). Bacterial diversity in the rhizosphere of Proteaceae species. *Environmental Microbiology*.

[14] Saitou N., Nei M. (1987). The neighbor-joining method: a new method for reconstructing phylogenetic trees. *Molecular Biology and Evolution*.

[15] Tamura K., Dudley J., Nei M., Kumar S. (2007). MEGA4: Molecular Evolutionary Genetics Analysis (MEGA) software version 4.0. *Molecular Biology and Evolution*.

[16] Jamieson A. D., Pruitt K. M., Caldwell R. C. (1969). An improved amylase assay. *Journal of Dental Research*.

[17] Gupta R., Gigras P., Mohapatra H., Goswami V. K., Chauhan B. (2003). Microbial *α*-amylases: a biotechnological perspective. *Process Biochemistry*.

[18] Kathiresan K., Manivannan S. (2006). *α*-amylase production by *Penicillium fellutanum* isolated from mangrove rhizosphere soil. *African Journal of Biotechnology*.

[19] Zaved H. K., Rahman M. M., Rahman A., Arafat S. M. Y., Rahman M. S. (2008). Isolation and characterization of effective bacteria for solid waste degradation for organic manure. *KMITL Science and Technology Journal*.

[20] Aneja K. R. (2003). *Experiments in Microbiology Plant Pathology and Biotechnology*.

[21] Felsenstein J. (1985). Confidence limits on phylogenies: an approach using the bootstrap. *Evolution*.

[22] Tamura K., Nei M., Kumar S. (2004). Prospects for inferring very large phylogenies by using the neighbor-joining method. *Proceedings of the National Academy of Sciences of the United States of America*.

[23] Naidub M. A., Saranraj P. (2013). Bacterial amylase: a review. *International Journal of Pharmaceutical and Biological Archive*.

[24] Oyeleke S. B., Oduwole A. A. (2009). Production of amylase by bacteria isolated from a cassava waste dumpsite in Minna, Niger State, Nigeria. *African Journal of Microbiology Research*.

[25] Khannous L., Jrad M., Dammak M. (2014). Isolation of a novel amylase and lipase-producing *Pseudomonas luteola* strain: study of amylase production conditions. *Lipids in Health and Disease*.

[26] Singh P., Gupta P., Singh R., Sharma R. (2012). Factors affecting alfa amylase production on submerged fermentation by *Bacillus* sp. *International Journal of Pharmacy and Life Sciences*.

[27] Raju E. V. N., Divakar G. (2013). Production of amylase by using *Pseudomonas aeruginosa* isolated from garden soil. *International Journal of Advances in Pharmacy, Biology and Chemistry*.

[28] Aiba S., Kitai K., Imanaka T. (1983). Cloning and expression of thermostable *α*-amylase gene from *Bacillus stearothermophilus* in *Bacillus stearothermophilu*s and *Bacillus subtilis*. *Applied and Environmental Microbiology*.

[29] Haki G. D., Rakshit S. K. (2003). Developments in industrially important thermostable enzymes: a review. *Bioresource Technology*.

[30] Samanta A., Mitra D., Roy S. N., Sinha C., Pal P. (2013). Characterization and optimization of amylase producing bacteria isolated from solid waste. *Journal of Environmental Protection*.

[31] Sivakumar T., Ramasubramanian V., Shankar T., Vijayabaskar P., Anandapandian K. T. K. (2001). Screening of keratinolytic bacteria *Bacillus cereus* from the feather dumping soil of sivakasi. *Journal of Basic and Applied Biology*.

[32] Haseltine C., Rolfsmeier M., Blum P. (1996). The glucose effect and regulation of alpha-amylase synthesis in the hyperthermophilic archaeon * Sulfolobus solfataricus*. *Journal of Bacteriology*.

[33] Linden A., Mayans O., Meyer-Klaucke W., Antranikian G., Wilmanns M. (2003). Differential regulation of a hyperthermophilic *α*-amylase with a novel (Ca,Zn) two-metal center by zinc. *The Journal of Biological Chemistry*.

[34] Burhan A., Nisa U., Gökhan C., Ömer C., Ashabil A., Osman G. (2003). Enzymatic properties of a novel thermostable, thermophilic, alkaline and chelator resistant amylase from an alkaliphilic *Bacillus* sp. isolate ANT-6. *Process Biochemistry*.

[35] Ramesh M. V., Lonsane B. K. (1989). Solid state fermentation for production of higher titres of thermostable alpha-amylase with two peaks for pH optima by Bacillus licheniformis M27. *Biotechnology Letters*.

[36] Lifshitz R., Levitzki A. (1976). Identity and properties of the chloride effector binding site in hog pancreatic *α*-amylase. *Biochemistry*.

[37] Saha K., Maity S., Roy S. (2014). Optimization of amylase production from *B. amyloliquefaciens*(MTCC 1270) using solid state fermentation. *International Journal of Microbiology*.

